# Development of Germline-Humanized Antibodies Neutralizing Botulinum Neurotoxin A and B

**DOI:** 10.1371/journal.pone.0161446

**Published:** 2016-08-25

**Authors:** Sebastian Miethe, Christelle Mazuet, Yvonne Liu, Robert Tierney, Christine Rasetti-Escargueil, Arnaud Avril, André Frenzel, Philippe Thullier, Thibaut Pelat, Remi Urbain, Alexandre Fontayne, Dorothea Sesardic, Michael Hust, Michel Robert Popoff

**Affiliations:** 1 Technische Universität Braunschweig, Institut für Biochemie, Biotechnologie und Bioinformatik, Abteilung Biotechnologie, Braunschweig, Germany; 2 Institut Pasteur, Unité des Bactéries anaérobies et Toxines, Paris, France; 3 National Institute for Biological Standards and Control (NIBSC), Division of Bacteriology, Potters Bar, United Kingdom; 4 Institut de Recherche Biomédicale des Armées (IRBA) Département des Maladies Infectieuses, Unité Interaction Hôte-Pathogène, Brétigny-sur-Orge, France; 5 LFB Biotechnologies, Therapeutic Innovation Department, Lille, France; Naval Research Laboratory, UNITED STATES

## Abstract

Botulinum neurotoxins (BoNTs) are counted among the most toxic substances known and are responsible for human botulism, a life-threatening disease characterized by flaccid muscle paralysis that occurs naturally by food poisoning or colonization of the gastrointestinal tract by BoNT-producing clostridia. To date, 7 serologically distinct serotypes of BoNT (serotype A-G) are known. Due to the high toxicity of BoNTs the Centers for Disease Control and Prevention (CDC) have classified BoNTs as category A agent, including the six biological agents with the highest potential risk of use as bioweapons. Well tolerated antibodies neutralizing BoNTs are required to deal with the potential risk. In a previous work, we described the development of scFv and scFv-Fc (Yumab) from macaque origin (*Macaca fascicularis*) neutralizing BoNT/A and B by targeting the heavy and light chain of each serotype. In the present study, we humanized the macaque antibodies SEM120-IIIC1 (anti-BoNT/A light chain), A1HC38 (anti-BoNT/A heavy chain), BLC3 (anti-BoNT/B light chain) and B2-7 (anti-BoNT/B heavy chain) by germline-humanization to obtain a better potential immunotolerance in humans. We increased the Germinality Index (GI) of SEM120-IIIC1 to 94.5%, for A1HC38, to 95% for BLC3 and to 94.4% for B2-7. Furthermore, the neutralization efficacies of the germline-humanized antibodies were analyzed in lethal and non-lethal *in vivo* mouse assays as full IgG. The germline-humanized IgGs hu8SEM120-IIIC1, hu8A1HC38, hu8BLC3 and hu8B2-7 were protective *in vivo*, when anti-heavy and anti-light chain antibodies were combined. The synergistic effect and high humanness of the selected IgGs makes them promising lead candidates for further clinical development.

## Introduction

Botulism is a life-threatening disease associated with foodborne poisoning caused by intoxication with botulinum neurotoxins (BoNTs) that are produced by the spore-forming bacteria *Clostridium botulinum* and certain other *Clostridium spp*. or by colonization of the gastrointestinal tract by BoNT-producing clostridia. An intoxication with BoNT is characterized by flaccid and life-threatening muscle paralysis that requires long term treatment in an intensive care unit [1,2]. BoNT are grouped in 7 serologically distinct serotypes (A to G). In 2014, a new serotype H was proposed [3,4], but further analysis indicates that it is a hybrid-like BoNT containing regions of similarity of BoNT/A1 and BoNT/F5 and was completely neutralized by serotype A antitoxin [5]. Four BoNT serotypes (A, B, E and rarely F) are responsible for most cases of human botulism [6]. Several cases of botulism, caused by BoNT/A, B and E, have been reported [7–11], but only 1% of all food poisoning-related cases of botulism, including adult toxin coinfections, are associated with BoNT/F [12]. BoNTs are type A-B heteromeric molecules first produced as 150 kDa single-chain protoxins, which are subsequently activated by proteolytic cleavage to generate a disulfide bond-linked structure containing a 50 kDa light chain and a 100 kDa heavy chain. The heavy chain contains two functional domains (HC and HN). These domains are required for toxin uptake into nerve cells by receptor-mediated endocytosis (HC) and for the translocation of the light chain across the membrane into the neuronal cytosol (HN) [13]. The toxicity of BoNTs is due to the catalytic domain of the light chain (a zinc endopeptidase), which cleaves specific components of the SNARE complex (soluble N-ethylmaleimide-sensitive factor attachment protein receptor) involved in the fusion of the synaptic vesicle with the presynaptic membrane, including VAMP (vesicle-associated membrane protein or synaptobrevin), SNAP25 (synaptosomal associated protein of 25 kDa) and Syntaxin [13]. BoNTs are classified as category A agents by the Centers for Disease Control and Prevention (CDC) due to their high toxicity. They belong to the group of six biological agents with the highest risk of potential to be used as bioweapons [14,15]. Iraq was weaponizing BoNTs during 1990/91 Gulf war and the Japanese cult Aum Shinrikyo attempted to use BoNT for bioterrorism [16,14,17]. In several scenarios, a risk deliberate contamination of the food supply chain by BoNTs has been highlighted [18]. The high biological relevance of BoNTs utilization as bioweapon requires the development of highly effective therapies. Antibodies represent significant drugs for the treatment of a multitude of pathogens and toxins. The current approach for treatment of botulism includes passive immunization with equine antitoxin sera consisting of Fab and/or F(ab')_2_ preparations [19]. Unfortunately, equine sera may cause serious adverse effects, including serum sickness and hypersensitivity [20]. This factor is particularly of concern for treatment of infant botulism. Therefore, in case of infant botulism human polyclonal anti-botulinum immunoglobulin preparations are used, such as BabyBig^®^ [21]. The quantity of these human preparations is limited and expensive [22]. The current situation illustrates the importance for new human-like or human antibodies that are highly effective and better tolerated than common equine antibody preparations or more available in case of human immunoglobulin preparations. In our previous studies, we described the development of neutralizing human-like scFv-Fc against the light and heavy chains of BoNT/A and B [23–25]. However, for future medical applications optimal tolerance in humans has to be ensured for these antibodies. Several humanization methods have been described [26]. One method especially for antibodies derived from non-human primates (NHP), called germline-humanization, is based on the modification of the NHP antibody framework regions (FR) to increase the level of identity with FRs encoded by the closest human germline gene sequences [27]. It is postulated that human germline FR, as part of IgM antibodies, are better tolerated by the immune system than FR sequences derived from IgG antibodies, which carry somatic hypermutations resulting from affinity maturation and probably forms immunogenic sequences [28,29]. Due to the high similarity of NHP and human antibodies, tools such as IMGT/V-QUEST can be used for identification of the human germline genes (V, (D), J), which are most similar to the sequence encoding by the NHP variable regions. Differences in the amino acid sequences are determined by the Germinality Index (GI) as a predictor of tolerance. The germline-humanization of 35PA83, an antibody of macaque origin neutralizing anthrax toxin in a pre-clinical development, led to an increase of the GI (from 87.6% to 97.7%), which is higher than GI of the fully human Fab 83K7C (GI 91.9%) used as a benchmark for this study [30]. Furthermore, the antibody WO-2 against Aβ peptide, associated with Alzheimer's disease, was successfully humanized by germline-humanization, retaining the affinity and ability to inhibit aggregation and oligomer-mediated toxicity [31].

In this article, we describe the germline-humanization of the four NHP antibodies SEM120-IIIC1 (anti-BoNT/A light chain) [24], A1HC38 (anti-BoNT/A heavy chain) [25], BLC3 (anti-BoNT/B light chain) and B2-7 (anti-BoNT/B heavy chain) [23] and their *in vivo* protection characteristics against BoNT/A and B when expressed as IgGs.

## Results

### Comparison between macaque anti-botulinum toxin antibodies and the most similar corresponding human germline genes

**Fig 1:**
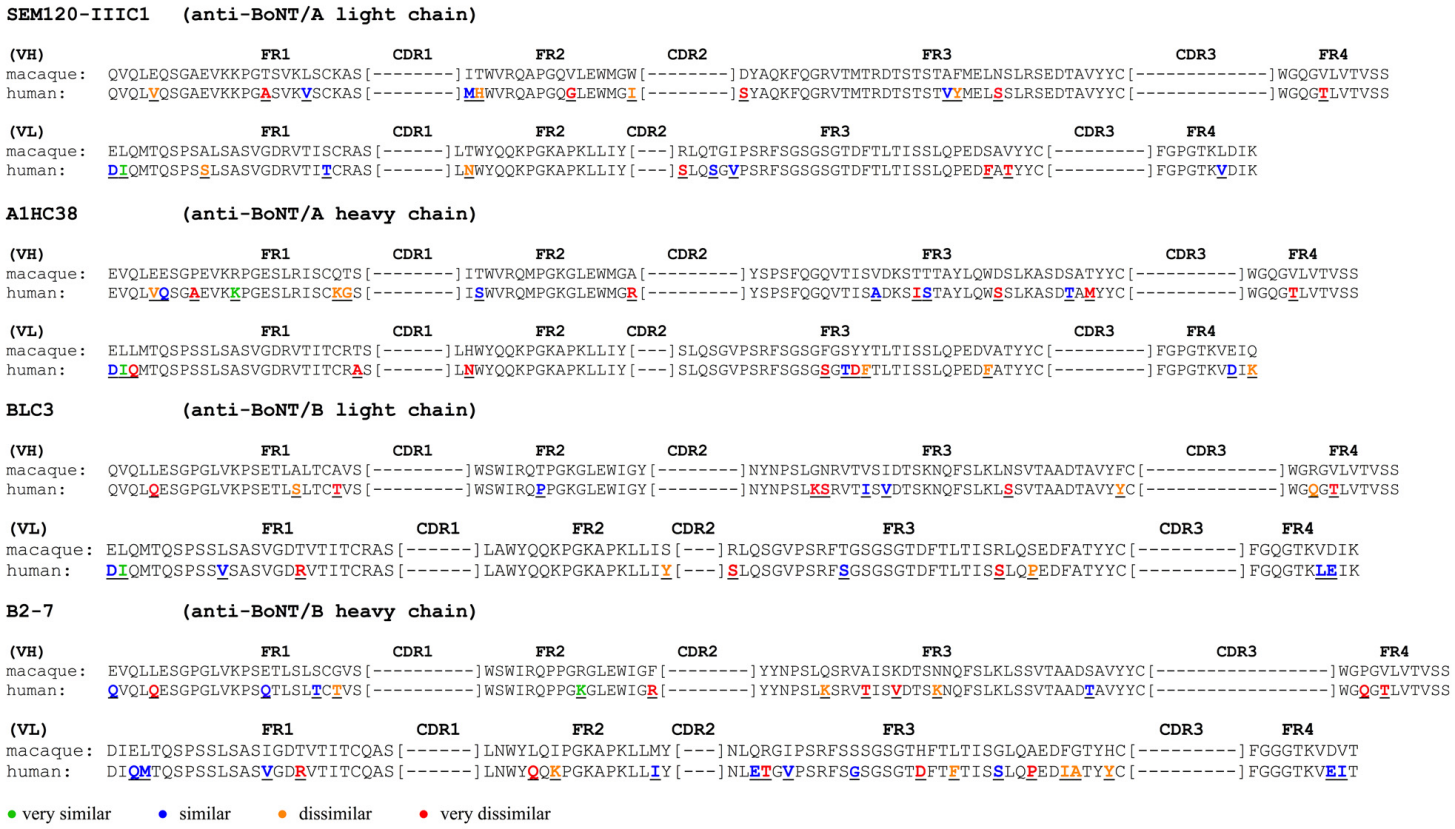
Sequence of the macaque framework regions and those coded by the most similar human germline genes. Based on the physiochemical classes of the amino acids (AA), differences in the framework regions are classified into very similar AA (green), similar AA (blue), dissimilar AA (orange) and very dissimilar AA (red).

In our previous studies, we reported the generation of neutralizing macaque scFv and scFv-Fc against BoNT/A and BoNT/B: SEM120-IIIC1 (anti-BoNT/A light chain), A1HC38 (anti-BoNT/A heavy chain), BLC3 (anti-BoNT/B light chain), B2-7 (anti-BoNT/B heavy chain) [23–25]. The comparison of the macaque VH and VL with the human germline genes was performed using IMGT/V-QUEST tool. The human germline genes most similar to the genes encoding the four anti-BoNT antibodies are given in Table 1. The Germinality Index (GI) for VH and VL of the macaque antibodies were calculated using IMGT/DomainGapAlign and provided an indication of the identity between framework regions of the antibodies and those encoded by the most similar human germline genes, as a percentage (Table 1). The differences of the amino acid (AA) sequence between SEM120-IIIC1, A1HC38, BLC3 and B2-7 framework regions and those coded by the most similar human germline genes were evaluated. In total, 23 AA (SEM120-IIIC1) and 27 AA (A1HC38) of the eight framework regions (180 AA) differed from those of the selected human germline gene segments. Twenty-three out of the 180 residues of the eight framework regions differed from BLC3 and those of the selected human germline gene segments. In the case of B2-7, 34 of the 179 residues of the FRs differed from the selected human germline gene segments with highest homology (Fig 1).

**Table 1:** Human germline genes most similar to the genes encoding the four anti-BoNT antibodies and the corresponding GI value.

Toxin		Antibody	VH			VL		GI [%]	
			V	D	J	V	J	VH	VL
A1	LC	SEM120-IIIC1	IGHV1-8*02	IGHD3-22*01	IGHJ4*02	IGKV1–39*01	IGKJ3*01	86.6	87.6
	HC	A1HC38	IGHV5-a*04	IGHD1-26*01	IGHJ5*02	IGKV1-39*01	IGKJ3*01	83.3	86.5
B2	LC	BLC3	IGHV4-28*06	IGHD5-12*01	IGHJ4*02	IGKV1-12*01	IGKJ2*01	86.8	87.6
	HC	B2-7	IGHV4-59*02	IGHD1-14*01	IGHJ5*02	IGKV1-33*01	IGKJ4*01	84.6	77.3

Based on the physicochemical classes of the amino acids, differences in the FRs were classified as very similar, similar, dissimilar and very dissimilar AA according to IMGT [32]. For VH of SEM120-IIIC1, we identified three residues classified as similar AA, four residues as dissimilar AA and five residues as very dissimilar AA. For VL, one residue was classified as very similar AA, five as similar AA, two as dissimilar AA and three as very dissimilar AA. In the case of A1HC38 (VH), we identified one residue classified as very similar AA, five as similar AA, three residues as dissimilar AA and six residues as very dissimilar AA. For VL, one residue was classified as very similar AA, three as similar AA, three as dissimilar AA and five as very dissimilar AA. For VH of BLC3, we identified three residues classified as similar AA, three as dissimilar AA and six as very dissimilar AA. For VL, one residue was classified as very similar AA, five as similar AA, two as dissimilar AA and three as very dissimilar AA. For VH of B2-7, one residue was identified as very similar AA, four as similar AA, three as dissimilar AA and six as very dissimilar AA. For VL, we identified ten residues as similar AA, five as dissimilar AA and five as very dissimilar AA (Fig 1).

### Germline-humanization of the macaque antibodies

**Fig 2:**
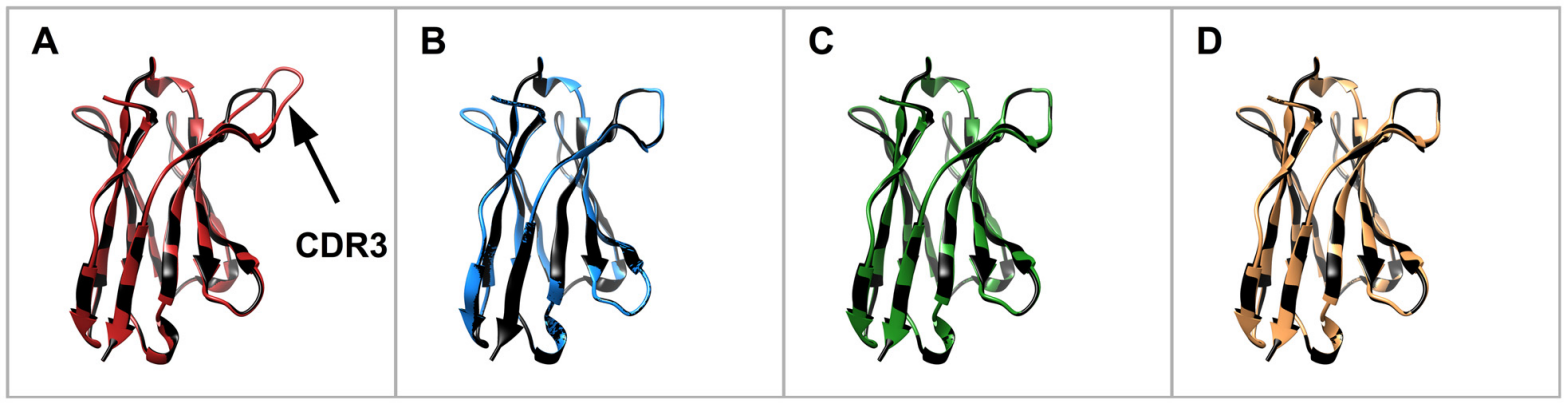
3D structure of the humanized VH variants. A) Comparison hu_1_VH (red) vs. SEM120-IIIC1 (black). B) Comparison hu_1_VH/V21>L (blue) vs. SEM120-IIIC1 (black). C) Comparison hu_2_VH/V21>L (green) vs. SEM120-IIIC1 (black). D) Comparison hu_3_VH/V21>L (orange) vs. SEM120-IIIC1 (black).

**Fig 3:**
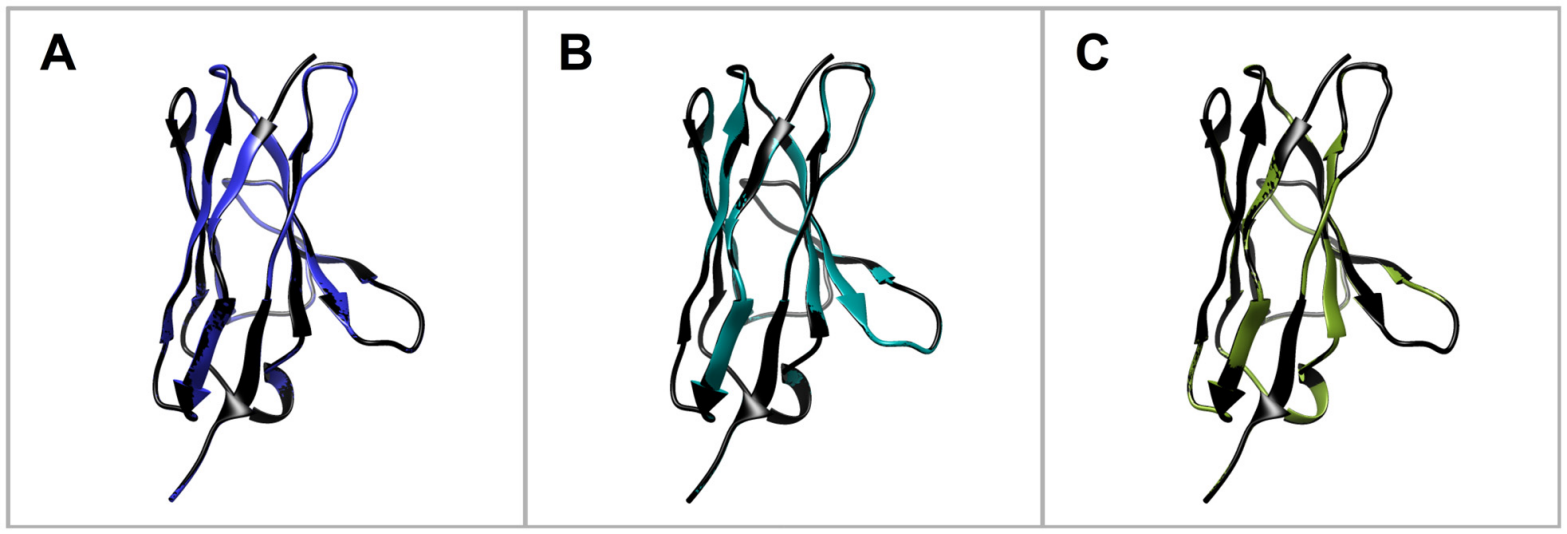
3D structure of the humanized VL variants. A) Comparison hu_1_VL (blue) vs. SEM120-IIIC1 (black). B) Comparison hu_2_VL (turquoise) vs. SEM120-IIIC1 (black). C) Comparison hu_3_VL (green) vs. SEM120-IIIC1 (black).

In a first step towards humanization we exchanged the AA in the FRs of SEM120-IIIC1, A1HC38, BLC3 and B2-7 with their human counterpart classified as very similar AA and similar AA. The resulting humanized variable domains were called hu_1_VH and hu_1_VL. Furthermore, we included the AA classified as dissimilar AA resulting in the humanized variants hu_2_VH and hu_2_VL. In the case of SEM120-IIIC1, we decided to exchange the AA classified as very dissimilar AA resulting in the humanized Variants hu_3_VH and hu_3_VL and modeled each variant using WAM antibody modeling [33]. The resulting structures of the respective humanized domains of SEM120-IIIC1 were compared with the determined parental structure of VH and VL (Figs 2 and 3). For VH, after exchange of the similar AA we observed a structural change of CDR3. Detailed analyses indicated that the exchange of leucine to valine in FR1 (V21>L) was responsible for this effect (Fig 2A and 2B). By retaining the parental AA at this position (hu_1_VH/V21>L) and further modifications including dissimilar AA and very dissimilar AA no significant changes of the structure were observed in the calculated structure model. In the next step, each humanized variable domain was combined with each other including the parental VH and VL and produced as scFv-Fc. The humanized variants of the resulting antibodies were termed hu1SEM120-IIIC1 up to hu16SEM120-IIIC1 (Table 2).

**Table 2:** GI value of the humanized variants of the different humanized variants of SEM120-IIIC1 and A1HC38.

Variant	VH Variant	VL Variant	Total GI
SEM120-IIIC1	macaque VH	macaque VL	87.2%
hu1SEM120-IIIC1	macaque VH	macaque VL	87.2%
hu2SEM120-IIIC1	macaque VH	hu_1_VL	90.6%
hu3SEM120-IIIC1	macaque VH	hu_2_VL	91.7%
hu4SEM120-IIIC1	macaque VH	hu_3_VL	93.4%
hu5SEM120-IIIC1	hu_1_VH/V21>L	macaque VL	88.3%
hu6SEM120-IIIC1	hu_1_VH/V21>L	hu_1_VL	91.7%
hu7SEM120-IIIC1	hu_1_VH/V21>L	hu_2_VL	92.8%
hu8SEM120-IIIC1	hu_1_VH/V21>L	hu_3_VL	94.5%
hu9SEM120-IIIC1	hu_2_VH/V21>L	macaque VL	90.5%
hu10SEM120-IIIC1	hu_2_VH/V21>L	hu_1_VL	93.9%
hu11SEM120-IIIC1	hu_2_VH/V21>L	hu_2_VL	94.9%
hu12SEM120-IIIC1	hu_2_VH/V21>L	hu_3_VL	96.7%
hu13SEM120-IIIC1	hu_3_VH/V21>L	macaque VL	93.3%
hu14SEM120-IIIC1	hu_3_VH/V21>L	hu_1_VL	96.7%
hu15SEM120-IIIC1	hu_3_VH/V21>L	hu_2_VL	97.8%
hu16SEM120-IIIC1	hu_3_VH/V21>L	hu_3_VL	99.5%
Variant	VH Variant	VL Variant	Total GI
A1HC38	macaque VH	macaque VL	84.4%
hu1A1HC38	macaque VH	hu_1_VL	87.4%
hu2A1HC38	macaque VH	hu_2_VL	89.1%
hu3A1HC38	hu_1_VH	macaque VL	88.3%
hu4A1HC38	hu_1_VH	hu_1_VL	90.5%
hu5A1HC38	hu_1_VH	hu_2_VL	92.2%
hu6A1HC38	hu_2_VH	macaque VL	89.9%
hu7A1HC38	hu_2_VH	hu_1_VL	92.2%
hu8A1HC38	hu_2_VH	hu_2_VL	94.9%

**Fig 4:**
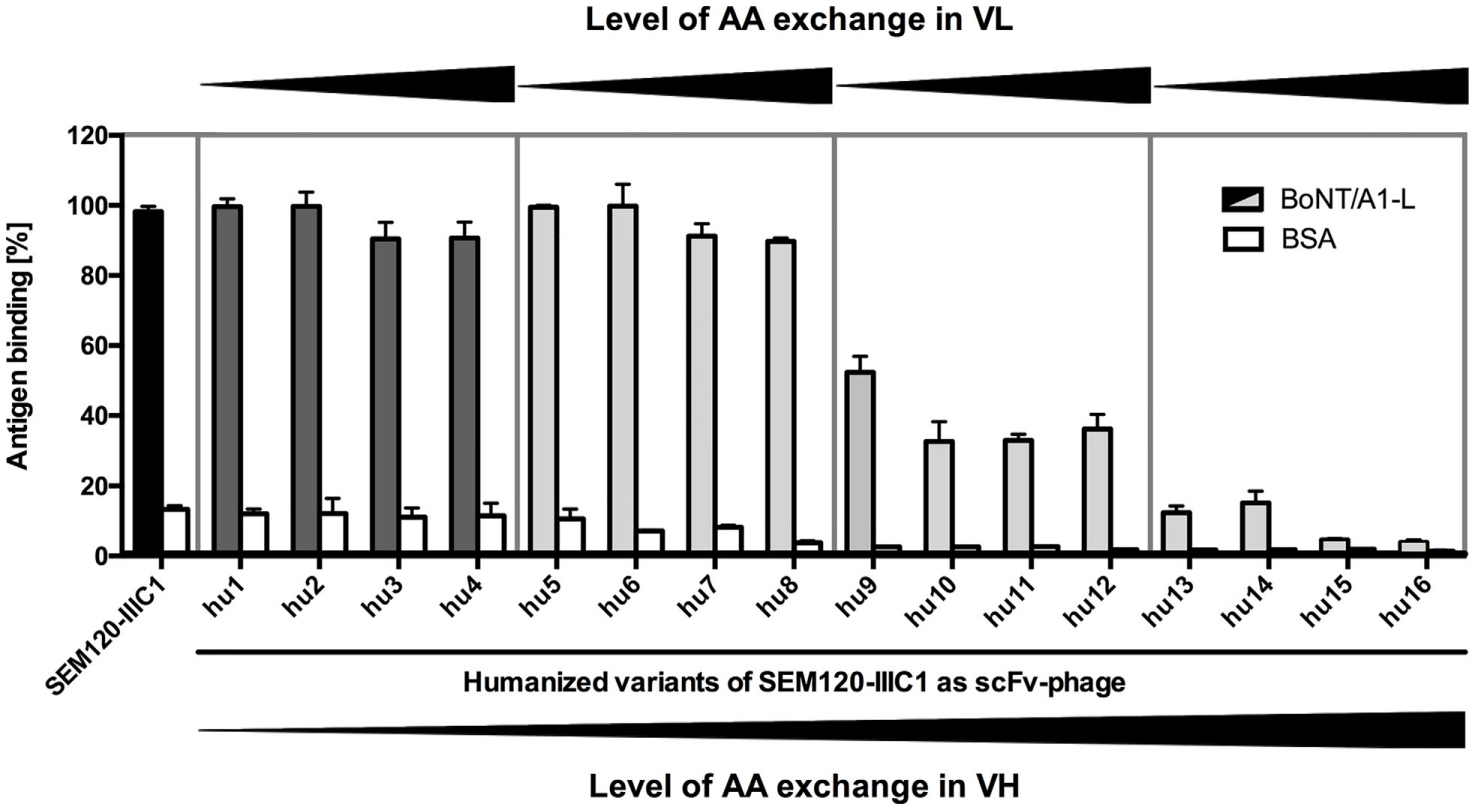
Antigen ELISA of the humanized variants of SEM120-IIIC1 against recombinant light chain of BoNT/A1. Binding of the germline-humanized anti-BoNT/A1 antibodies (hu1-hu16SEM120-IIIC1) as scFv-Fc (each 1 μg) was tested on 100 ng recombinant BoNT/A1 light chain.

The antigen binding of the 16 humanized variants was compared and validated by ELISA using immobilized recombinant light or heavy chain of BoNT and by surface plasmon resonance (SPR) analyses using holotoxin. Based on the results of the ELISA and SPR analyses we selected hu8SEM120-IIIC1 for further *in vivo* studies (Fig 4, Table 3). This humanized antibody with a total GI value of 94.5% was generated by adapting the 4 FR regions of the light chain to the most similar human germline genes, resulting in a GI value of 100% for VL. For VH (GI 89%), only 2 similar AAs were exchanged compared to the most similar human germline genes. Hu8SEM120-IIIC1 has nearly the same affinity (1.41 nM) against the holotoxin compared to the parental antibody (0.82 nM). The exchange of the dissimilar AA and very dissimilar AAs in the heavy chain of SEM120-IIIC1 led to a reduction of antigen binding. The effect of the AAs which were not exchanged in hu8SEM120-IIIC1 were observed by single back-mutations and tested by ELISA neutralization in the mouse phrenic nerve-hemdiaphragm *ex vivo* studies [24]. The mutation of leucine to valine in FR1 (V21>L) resulted in reduction of toxin neutralization efficiency (data not shown) compared to hu8SEM120-IIIC1 which is in accordance with the structure model. Furthermore, single mutations of dissimilar AA or very dissimilar AAs located in VH reduced the antigen binding of the humanized anti-BoNT/A light chain antibody.

**Table 3:** Affinity measurement of the humanized SEM120-IIIC1 variants as scFv-Fc against holotoxin BoNT/A1 (no affinities for hu9-hu16, no reactivity).

variant	k_off_ [s^-1^]	k_on_ [M^-1^s^-1^]	K_D_ [nM]
SEM120-IIIC1	6.87 x 10^−5^	8.34 x 10^4^	0.82
hu1SEM120-IIIC1	1.14 x 10^−4^	1.09 x 10^5^	1.04
hu2SEM120-IIIC1	1.07 x 10^−4^	1.06 x 10^5^	1.02
hu3SEM120-IIIC1	2.14 x 10^−4^	1.07 x 10^5^	1.99
hu4SEM120-IIIC1	1.48 x 10^−4^	1.64 x 10^5^	0.89
hu5SEM120-IIIC1	1.09 x 10^−4^	6.35 x 10^4^	1.71
hu6SEM120-IIIC1	8.31 x 10^−5^	5.73 x 10^4^	1.45
hu7SEM120-IIIC1	2.27 x 10^−4^	1.18 x 10^5^	1.92
hu8SEM120-IIIC1	1.33 x 10^−4^	9.41 x 10^4^	1.41

Based on the results of the humanization of SEM120-IIIC1, for the germline-humanization of A1HC38, BLC3 and B2-7 we only used very similar, similar and dissimilar AA exchanges. The resulting humanized antibody variants were named hu1A1HC38 up to hu8A1HC38, hu1BLC3 up to hu8BLC3 or hu1B2-7 up to hu8B2-7 (Table 4). The antigen binding of the eight humanized variants of A1HC38, BLC3 and B2-7 was compared and validated by ELISA using recombinant light and heavy chain of BoNT/A or B (S1, S2 and S3 Figs). Based on these results, we decided to use hu8A1HC38, hu8BLC3 and hu8B2-7 variants for further *in vivo* studies.

**Table 4:** GI value of the humanized variants of the different humanized variants of BLC7 and B2-7.

Variant	VH Variant	VL Variant	Total GI
BLC3	macaque VH	macaque VL	87.2%
hu1BLC3	macaque VH	hu_1_VL	90.6%
hu2BLC3	macaque VH	hu_2_VL	91.7%
hu3BLC3	hu_1_VH	macaque VL	88.9%
hu4BLC3	hu_1_VH	hu_1_VL	92.3%
hu5BLC3	hu_1_VH	hu_2_VL	93.4%
hu6BLC3	hu_2_VH	macaque VL	90.5%
hu7BLC3	hu_2_VH	hu_1_VL	93.9%
hu8BLC3	hu_2_VH	hu_2_VL	95.0%
Variant	VH Variant	VL Variant	Total GI
B2-7	macaque VH	macaque VL	81.0%
hu1B2-7	macaque VH	hu_1_VL	86.1%
hu2B2-7	macaque VH	hu_2_VL	89.5%
hu3B2-7	hu_1_VH	macaque VL	83.7%
hu4B2-7	hu_1_VH	hu_1_VL	88.8%
hu5B2-7	hu_1_VH	hu_2_VL	92.2%
hu6B2-7	hu_2_VH	macaque VL	85.9%
hu7B2-7	hu_2_VH	hu_1_VL	91.0%
hu8B2-7	hu_2_VH	hu_2_VL	94.4%

By performing germline-humanization we increased the GI of SEM120-IIIC1 to 94.5% and to 94.9% for A1HC38, to 95% for BLC3 and to 94.4% for B2-7 (Tables 2 and 4).

### Production of germline-humanized IgG

The full length cDNA coding each pair of heavy and light chains were obtained by fusing by PCR assembly V_H_ and V_K_ with the human IgG1 and κ constant regions respectively. These sequences were then introduced into the expression vector HKgenEFss by Infusion. The YB2/0 cell line was transfected with the resulting constructs to generate stable pools for production in roller bottles. The culture supernatants were harvested, concentrated and filtered. The antibodies were then purified by affinity chromatography on Sepharose protein A followed by sterile filtration. The purified antibodies were considered pure by SDS-PAGE. Aggregates and endotoxin contaminations were monitored by SEC and LAL assay respectively. The detected quantities were below 5 IU/mg of endotoxin and 0.7% of aggregates making the use of these antibodies compatible with *in vivo* evaluation.

### *In vivo* protection (paralysis assays)

**Fig 5:**
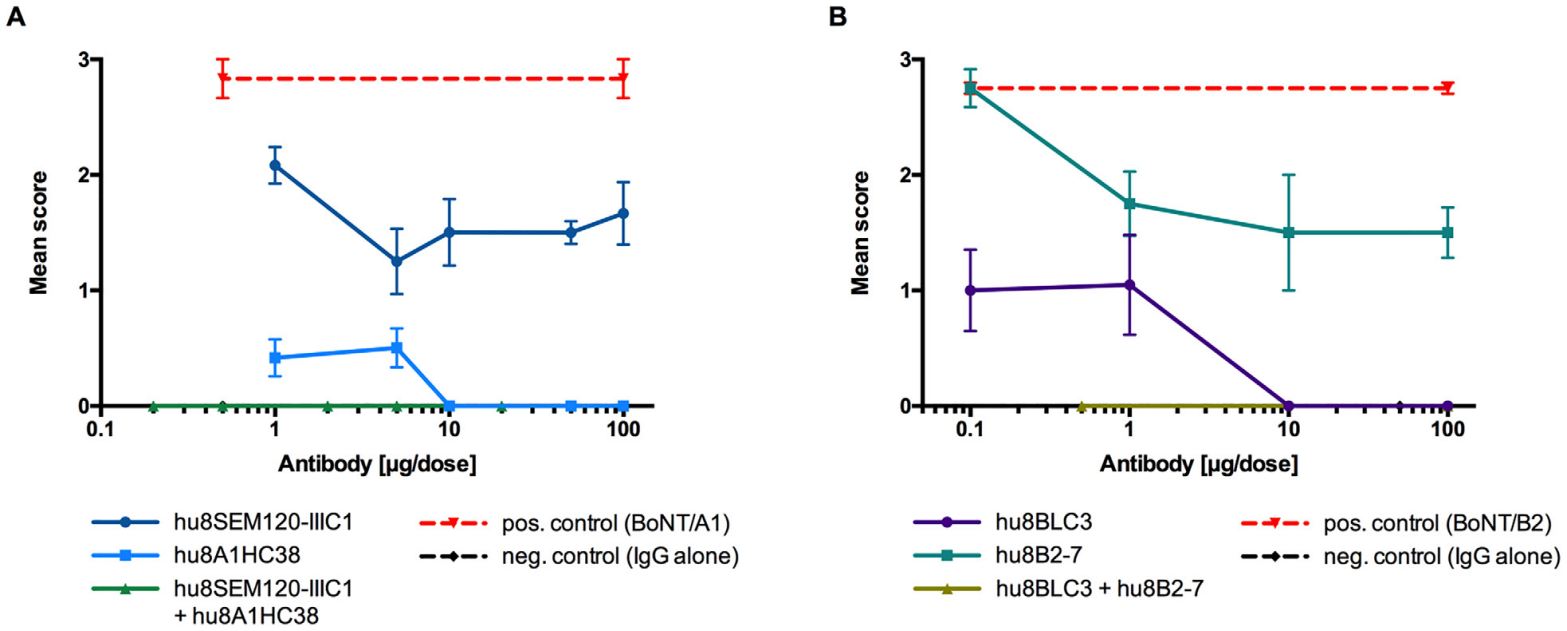
Protection capacities of IgGs. *In vivo* mouse paralysis assay was performed to determine protection capacities of IgGs targeting BoNT/A (5A) or BoNT/B (5B) light and heavy chains. A) Pure BoNT/A1 (0.4 LD_50_ per dose, 1.74 pg) was pre-mixed with either hu8SEM120-IIIC1 (light chain) or hu8A1HC38 (heavy chain) at 100 μg, 50 μg, 10 μg, 5 μg or 1 μg of antibody per dose. When tested in combination IgGs (hu8SEM120-IIIC1 and hu8A1HC38 50) were pre-mixed with the same concentration of BoNT/A1 and 50 μg, 10 μg, 2.5 μg, 1.0 μg, 0.25 μg or 0.1 μg of each antibody per dose (100 μg, 20 μg, 5.0 μg, 2.0 μg, 0.5 μg or 0.2 μg total IgG). B) Complex BoNT/B2 (0.2 LD_50_ per dose) was pre-mixed with either hu8BLC3 (light chain) or hu8B2-7 (heavy chain) at 100 μg, 10 μg, 1 μg or 0.1 μg per dose. When tested in combination IgGs (hu8BLC3 and hu8B2-7) were pre-mixed with the same concentration of BoNT/B2 and 50 μg or 0.25 μg of each antibody per dose (100 μg or 0.5 μg total IgG). In both studies mixtures of toxin and antibody were left for 30 min at room temperature before 0.1 mL was injected subcutaneously into left inguinocrural region of female MF1 strain of mice (n = 4). Animals were scored at 48 hr post injection. Results are expressed as mean score of 4 mice ± SEM. Positive control group of mice received a single injection of either BoNT/A1 (0.4 LD_50_ per dose) or BoNT/B2 (0.2 LD_50_ per dose). In each study negative control comprised of one group of mice (n = 4) injected with the highest concentration of IgG mixtures (50 μg each) without toxin (data not shown).

The selected IgGs targeting BoNT/A light chain (hu8SEM120-IIIC1) and heavy chain (hu8A1HC38) were tested *in vivo* in the mouse flaccid paralysis protection model both individually, and in combination for synergistic effect against pure BoNT/A1 (0.4 LD_50_ per dose, 1.74 pg) toxin. Results from observation of animals after 48 h are presented in Fig 5A. Complete protection was achieved with 100 μg, 50 μg and 10 μg per dose of hu8A1HC38, targeting the heavy chain of BoNT/A1 toxin, whereas partial protection was observed at all lower doses tested (5 μg and 1 μg per dose). However, hu8SEM120-IIIC1, targeting the light chain of BoNT/A1 did not fully protect against the paralysis induced by BoNT/A1 toxin, even at 100 μg of IgG per dose (Fig 5A). In fact all doses of antibody between 100 μg and 5 μg provided similar protective effect with no benefit of dose increase. Additional reduction in protection was seen when the dose of IgG was further reduced to 1 μg per dose. When these two IgGs, were combined as mixture (hu8SEM120-IIIC1 and hu8A1HC38) full protection was observed at all doses used. The lowest dose where animals remained fully protected from paralysis was 0.1 μg of each IgG (0.2 μg total IgG), whereas full protection was not observed at 1.0 μg of individual antibody, confirming synergistic effect (Fig 5A).

The selected IgGs targeting the BoNT/B light chain (hu8BLC3) and heavy chain (hu8B2-7) were also tested individually and in combination for synergistic effect against paralysis induced by complex BoNT/B2 (0.2 LD_50_ per dose). Results for 48 hr after intoxication are presented in Fig 5B. Complete protection was achieved with hu8BLC3 at 100 μg, 50 μg and 10 μg per dose and partial protection with two further 10-fold dilutions (Fig 5B). Antibody hu8B2-7 did not significantly protect mice at any of the concentrations used in this study from 100 μg to 0.1 μg per dose. However, the combination of hu8B2-7 and hu8BCL3 fully protected mice *in vivo* at 0.25 μg dose of each IgG (0.5 μg total IgG) where protection was not observed for each antibody alone at 1.0 μg dose.

Positive control group of mice received a single injection of either BoNT/A1 (0.4 LD_50_ per dose) or BoNT/B2 (0.2 LD_50_ per dose) where a mean score of approximately 3 was observed in both studies (Fig 5). Negative control group of mice (n = 4) was injected with 100 μg of IgG mixtures (50 μg of each IgG) in the absence of toxin and did not show any local effect (data not shown).

### *In vivo* protection (lethal assays)

To further investigate the *in vivo* neutralizing potency of the antibodies, the selected IgGs targeting BoNT/A light chain (hu8SEM120-IIIC1) and heavy chain (hu8A1HC38) were tested *in vivo* mouse lethality both individually and in combination for synergistic effect against complexed form of BoNT/A1 using 5x mouse 50% lethal doses (5 MLD_50_) per mouse (Table 5). Although partial protection was achieved with 25 μg and 2.5 μg of hu8SEM120-IIICI or 2.5 μg of hu8A1HC38, respectively, complete neutralization of BoNT/A1 lethal activity was obtained by combining both antibodies. The lowest fully protective dose was 2.5 μg of each IgG (5 μg total IgG), whereas 25 μg of individual antibody only induced a partial protection or survival, but with strong symptoms of botulism intoxication (hu8A1HC38), thus confirming the synergistic effect between the two antibodies (Table 5). When the dose was further reduced tenfold (0.25 μg of each IgG), 50% protection in mice was still observed (Table 5). The selected IgGs targeting the BoNT/B light chain (hu8BLC3) and heavy chain (hu8B2-7) were also tested individually and in combination for synergistic effect against lethality induced by complex BoNT/B2 (approximately 5 MLD_50_/mouse). The results for BoNT/B2 were quite similar to those obtained with antibodies against BoNT/A1 (Table 6): partial protection was achieved with doses of 25 μg and 2.5 μg of hu8BLC3 (anti-BoNT/B light chain) and no protection or extremely weak protection with the same doses of hu8B2-7 (anti-BoNT/B heavy chain) tested individually. However, the combination of hu8B2-7 and hu8BLC3 fully protected mice *in vivo* at 2.5 μg dose of each IgG (5 μg total IgG). Further reduced doses (0.25 μg of each IgG) still induced 50% protection against 5 MLD_50_ of BoNT/B2 (Table 6).

**Table 5:** Protection capacities of IgGs in *in vivo* mouse assay. Neutralization potencies of hu8SEM120-IIIC1 and hu8A1HC38, tested individually or in combination, against 5 MLD_50_ of BoNT/A1 in complex form. The preparations of antibodies and toxin (0.5 mL) were injected intraperitoneally into Swiss mice (20–22 g). The results are expressed as the number of the surviving mice of the total number of mice injected.

Antibody	BoNT/A1 (strain HALL)		
	5 MLD_50_/mice (survival mice/total mice)		
	hu8SEM120-IIIC1	hu8A1HC38	Combination
25 μg/mice	1/4	4/4 (4 very symptomatic)	8/8
2.5 μg/mice	3/4	2/4	8/8
0.25 μg/mice	0/4	1/4	4/8

**Table 6:** Protection capacities of IgGs in *in vivo* mouse assay. Neutralization potencies of hu8BLC3 and hu8B2-7, tested individually or in combination, against 5 MLD_50_ of BoNT/B2 in complex form. The preparations of antibodies and toxin (0.5 mL) were injected intraperitoneally into Swiss mice (20–22 g). The results are expressed as the number of the surviving mice of the total number of mice injected.

Antibody	BoNT/B2 (strain BL6, complexed form)		
	5 MLD_50_/mice (survival mice/total mice)		
	hu8BLC3	hu8B2-7	Combination
25 μg/mice	6/8	1/4	8/8
2.5 μg/mice	5/8	0/4	8/8
0.25 μg/mice	0/4	2/4	5/8

## Discussion

In previous studies, neutralizing macaque antibodies were generated against BoNT/A. The anti-BoNT/A1 antibody SEM120-IIIC1 isolated from a macaque immune library inhibited the endopeptidase activity of BoNT/A1 *in vitro* with a molar ratio of 5:1 (antibody binding:toxin) as scFv-Fc and neutralized BoNT/A1 toxicity in the *ex vivo* mouse phrenic nerve-hemidiaphragm assay by targeting the light chain of BoNT/A1 [24]. The scFv A1HC38 targeting the heavy chain of BoNT/A1 was highly effective in the *ex vivo* mouse phrenic nerve-hemidiaphragm assay, that mimics the *in vivo* respiratory paralysis caused by BoNTs [25]. The antibodies BLC3 and B2-7 respectively targeting the light and heavy chain of BoNT/B2, were isolated from immune libraries. The scFv-Fc (Yumab) format of these antibodies neutralized BoNT/B2 toxicity in the *ex vivo* mouse phrenic nerve-hemidiaphragm assay. Both scFv-Fc in combination were highly effective in the *in vivo* mouse flaccid paralysis assay, where complete protection against BoNT/B2 (0.2 LD_50_ p**e**r mouse) was achieved [23]. The high “humanness” of all four antibodies predicts a high tolerance in human. Studies with the chimeric antibody lumiliximab, consisting of the variable regions of a macaque in combination with the human constant regions, showed a good immune tolerance in human subjects [34,35]. Nevertheless, the germline-humanization of these antibodies provides a potentially promising method for increasing immune tolerance for human treatment [27]. This method was successfully used for the humanization of 35PA38, an antibody neutralizing the anthrax lethal toxin isolated from a macaque immune library which is currently in clinical development as IgG, where GI value was increased to 97.8% [30]. For comparison, the average GI value of 95.7% was calculated for 500 scFvs isolated from the human naïve antibody gene library HAL7/8 [36]. The number of differences between the framework regions of the macaque SEM120-IIIC1 and those encoded by the closest human germline genes was 23, resulting in a GI value of 87.2%, whereas 27 different AA were identified in the framework regions of A1HC38 resulting in a GI value of 84.4%. For the anti-BoNT/B antibodies, 23 AA (BLC3) and 34 AA (B2-7) were identified in the FRs that differs from the closest human germline genes, resulting in a GI value of 87.2% (BLC3) and 81% (B2-7).

These differences could either correspond to somatic hypermutations or to differences between the macaque and human germline genes and both could be immunogenic. Therefore, we decided to increase the GI value by a systematic approach. We adapted the FRs of SEM120-IIIC1, A1HC38, BLC3 and B2-7 to the human counterparts, because the human germline FRs as part of IgM antibodies should be as well tolerated as any other human protein, in contrast to the FR sequences derived from IgG antibodies, which carry somatic hypermutations resulting from affinity maturation and probably forms immunogenic sequences [29,37]. For the humanization process dedicated to decrease immunogenicity of the four antibodies we used a multistep approach. In the first step, we designed humanized variants of the variable domains by exchanging the AA in the FRs that differ from the human germline sequence with their human counterpart classified as very similar and similar AA. The resulting variable domains were called hu_1_VH and hu_1_VL. In the next step we included the AA classified as dissimilar AA, resulting in the humanized variants hu_2_VH and hu_2_VL and combined each variable domain with each other including the parental VH and VL. In the case of SEM120-IIIC1 we additionally exchanged the very dissimilar AA (hu_3_VH and hu_3_VL). By exchanging these AA, we were able to increase the GI value of the humanized antibodies up to 94.5% (hu8SEM120-IIIC1), 94.9% (A1HC38), 95% (hu8BLC3) and 94.4% (hu8B2-7).

For the humanized variants, only ten (hu8SEM120-IIIC1), eleven (hu8A1HC38), nine (hu8BLC3) and eleven (hu8B2-7) AA of the parental antibody were retained in the frameworks. The average GI of 500 scFv, isolated out of the naïve human antibody gene library HAL7/8, was 96.8% (VH), 95.4% (VL lambda) and 94.8% (VL kappa). With GI values of 95% (hu8BLC3), 94.4% (hu8B2-7), 94.5% (hu8SEM120-IIIC1) and 94.9% (hu8AHC38) the germline-humanized variable domains are as human as naïve human germline derived variable domains.

The protective capacities of the four germline-humanized IgG, targeting the light and heavy chains of BoNT/A and BoNT/B were evaluated initially in the mouse local flaccid paralysis assay, which provides a less time consuming and more humane alternative to the LD_50_ assay [38]. Evaluating for protection of mice against locally induced toxin effect was considered a first step towards validation of protection capacities of selected IgGs. Whereas scFv-Fc against BoNT/B were previously tested in mouse paralysis assay *in vivo* [23], similar studies were not performed with scFv or scFv-Fc targeting BoNT/A light [24] or heavy chains [25], which were only evaluated for toxin neutralization properties in the mouse phrenic nerve-hemidiaphragm assay *ex vivo* on tissue isolated from mice. The studies confirmed that IgG hu8A1HC38 at 10 μg per dose fully protected mice from paralysis whereas hu8SEM120-IIIC1 did not protect, even at 100 μg dose. In combination, these two antibodies at as low as 0.1 μg each (0.2 μg total IgG) per dose fully protected mice from paralysis induced by 0.4 LD_50_, (1.74 pg) of toxin which increased the ratio of protection by at least 100-fold.

In agreement to our previous observation using scFv-Fc preparations of BLC3 and B2-7 [23], we confirmed that IgG hu8B2-7 alone, even at 100 μg, did not protect mice from paralysis induced by 0.2 LD_50_ of BoNT/B2, whereas IgG hu8BLC3 at 10 μg did confer protection.

In case of the anti-BoNT/B2 antibodies we made an interesting observation. We expected that the main protective effect against BoNTs based on the binding of the heavy chain, which is responsible for receptor binding and translocation of the light chain into the cytoplasm. The protective effect of the anti-light chain antibody hu8BLC3 could be caused by binding an epitope close to the heavy chain that interacts with receptor binding or inhibits the translocation of the light chain into the cytoplasm. It is also possible that hu8BLC3 still blocking the endopeptidase activity of the light chain in the cytoplasm.

Furthermore, in agreement to previous observations with scFv-Fc, combination of IgG also exhibited synergistic protection in mice when each IgG was given at 0.25 μg per dose (0.5 μg total IgG). Results obtained in the mouse lethality test corroborated neutralization property of individual selected antibodies obtained in the mouse phrenic nerve- hemi diaphragm *ex vivo* assay and/or in the local flaccid paralysis mouse assay [23–25]. Where a partial protection was achieved with doses of 25 μg and 2.5 μg of anti-BoNT/A light chain (hu8SEM120-IIIC1) or anti-BoNT/A heavy chain (hu8A1HC38) tested individually, complete neutralization of the lethal activity induced by 5 MLD_50_ of BoNT/A1 was obtained by combining the two antibodies at 2.5 μg each (5 μg total IgG). Similarly, we observed a protective synergistic effect of antibodies directed against the heavy chain (hu8B2-7) and light chain (hu8BLC3) of BoNT/B down to the dose of 2.5 μg (5 μg total IgG against 5 MLD_50_ BoNT/B). Both lethal and paralysis assays in mice established that full protection against 1 LD_50_ of either BoNT/A or BoNT/B can be achieved with 1 μg of two IgGs in combination. The demonstration of the protective synergistic effect is in agreement with results obtained with an oligoclonal recombinant antibody preparation composed of 3 mAbs directed against BoNT/A [39] and with the combination of two mAbs directed against BoNT/A HC and the LC domains [40]. However, single mAbs or recombinant chimeric counterparts have been proved to be very efficient in neutralizing BoNT/A activity in the mouse protection assay without additional antibodies [41–43]. A combination of the four germline-humanized anti-BoNT/A and B IgGs with a formerly described anti-BoNT/E antibody [44] would result in a promising oligoclonal antibody product that could be effective against the three most important BoNTs.

In combination of the germline-humanized IgG targeting the HC and LC domain of BoNT/A and B are neutralizing *in vivo* and it is expected that these IgGs are well tolerated in humans with less or no adverse effect. In contrast to usual therapeutics, such as BabyBig^®^ or HBAT, they can be produced in large amount without the use of animals and are suitable for further clinical development as part of an oligoclonal drug for treatment of botulism. Such a drug would be helpful both for the European Union biodefense and for the treatment of natural botulism.

## Materials and Methods

### Ethical statements and animal care

The *in vivo* local flaccid paralysis assay was performed at NIBSC by an approved procedure covered by the UK Home Office project license (PPL#80/2634, granted to Dr. Sesardic) which covered research under AntiBotABE project. The procedure involves administration subcutaneously into mice of sub-lethal doses of BoNT premixed with antibody for neutralization studies, and observation for signs of abdominal ptosis with local palsy in mice over a period of 48 hr at the site of injection. None of the animals experience systemic botulinum toxicity in this procedure [38]. All such experiments comply with the UK Home Office regulations for the use of animals in research under Animals (Scientific Procedures) Act 1986 (ASPA) and revised European Directive 2010/63 EU on the protection of animals. Experiments performed at NIBSC for this study were approved by the local animal research oversight committee AWERB (Animal Welfare and Ethics Review body).

The *in vivo* mouse lethality test was performed at Institut Pasteur in accordance with French and European Community guidelines for laboratory animal handling. The protocols of experiments were approved by the Institut Pasteur (Agreement of laboratory animal use n° 2013–0118). Mice were kept at a constant temperature (22°C ±2°C) and relative humidity (50%), with 12 hr of artificial light per day. They were housed in individual cages (8 per room). Mice were fed with dried food and water at libitum. Mice were observed every two hours post-injection except during the night from 7 pm to 7 am, over a period of 4 days. The animals with characteristic symptoms of botulism including muscle paralysis, respiratory difficulty were euthanized by cervical dislocation. No unexpected death (death in non symptomatic animals) was observed.

### Humanization of the macaque antibodies by germline-humanization

The sequence of each antibody was compared with the human germline genes using the IMGT/V-QUEST online tool from the International ImmunoGeneTics information system^®^ (IMGT) (http://www.imgt.org). This tool allows identification of the human germline genes most similar to any given variable region and calculates the Germinality Index (GI), defined as the percentage identity between a given framework region (FR) and the most similar human germline sequence. Based on the physiochemical classes of the amino acids (AA), differences in the FRs were classified as very similar, similar, dissimilar and very dissimilar AA. Firstly, humanized variants of VH and VL were designed by exchanging the very similar AA and similar AA followed by dissimilar AA and very dissimilar AA. The structure of each domain was modeled using WAM antibody modeling [33] and visualized using UCSF Chimera software [45]. The humanized VH and VL genes were synthesized by GeneArt^®^ Gene Synthesis (Regensburg, Germany) and were used for expression of scFv-Fc and IgG.

### Production and purification of scFv-Fc antibodies

All humanized variants were subcloned into pCSE2.5-mIgG2c-Fc-XP and produced as scFv-Fc antibody in HEK293-6E cells (National Research Council (NRC), Biotechnological Research Institute (BRI)) cultured in chemically defined medium F17 (Thermo Fisher Scientifics, Waltham, USA) supplemented with 1 g/L pluronic F68 (Applichem, Darmstadt, Germany), 4 mM L-glutamine (Biochrom GmbH, Berlin, Germany) and 25 mg/L G418 (Biochrom GmbH, Berlin, Germany), as previously described [46]. The scFv-Fc produced were chimeric macaque-mouse antibodies. DNA was used for the transient transfection of 25 mL cultures of HEK293-6E cells in 125 mL Erlenmeyer shake flasks. After 48 hr of culture with shaking at 110 rpm in a Minitron orbital shaker (Infors GmbH, Einsbach, Germany) at 37°C, under an atmosphere containing 5% CO2, one volume of culture medium, with a final concentration of 0.5% (w/v) tryptone N1 (TN1, Organotechnie S.A.S., La Courneuve, France), scFv-Fc were purified on a UNOsphere SUPrA column (Biorad, Hercules, USA) with a Profinia apparatus (Biorad, Hercules, USA), according to the manufacturer’s instructions.

### Cloning of germline-humanized IgG

The different germline-humanized IgGs were constructed and expressed as described previously [47]. Briefly, the DNA sequences for variable and constant regions for heavy and light chains were obtained by PCR and sub-cloned sequentially by Infusion into HKgenEFss vector. This generic vector is developed for an optimal expression into the rat myeloma cell line YB2/0. Bacterial transformation was performed in *E*. *coli* Top10 cells (Thermo Fisher Scientifics, Waltham, USA) for each plasmid ligation in order to select the ligated product and amplification. Positive clones were selected by PCR and digestion on individual colonies was performed. Subsequently, colonies were grown overnight at 37°C in LB medium with ampicillin. Plasmids were isolated using the NucleoSpin kit (Macherey-Nagel, Berlin, Germany), and inserted sequences were verified (MWG, Ebersberg, Germany).

### Production and purification of germline-humanized IgG

The stable expression of the humanized antibody was obtained as previously described [47]. Briefly, YB2/0 were stably transfected with the linearized expression vectors. The humanized IgGs were produced in YB2/0 over 5 to 7 days using EMS (Invitrogen, Carlsbad, USA), 5% Ultra low IgG FCS (PAA) and 0.5 g/L G418. The antibodies were purified from culture supernatant by affinity chromatography onto protein A sepharose (GE-Healthcare, Chalfont St Giles, UK). The level of aggregates and endotoxins were determined by gel filtration on Superdex HR/200 (GE-Healthcare, Chalfont St Giles, UK) and by LAL (limulous amoebocyte lysate) testing [48], respectively. Antibody quality and purity was also monitored by SDS-PAGE and Coomassie staining. In addition, glycosylation patterns and the core fucose percentage were determined for each purified antibody by high performance capillary electrophoresis laser induced fluorescence (HPCE-Lif) [49,50] confirming the EMABling characteristic.

### ELISA analyses

ELISA was performed in 96-well microtiter plates (Corning, New York, USA). Each well was coated with recombinant light or heavy chain of BoNT/A or B (100 ng per well) (ListLabs, Campbell, UK), in 100 μL of phosphate-buffered saline (PBS) by incubating overnight at 4°C. The coated wells were washed three times with PBST (PBS + 0.05% Tween 20) using an ELISA plate washer (Tecan Columbus, Tecan, Männedorf, Switzerland). The wells were then blocked by incubation with 2% (w/v) skimmed milk powder in PBS supplemented with 0.1% Tween 20 (2% M-PBST) for 1 hr at room temperature, and then washed three times with PBST. For the antigen ELISA with scFv-Fc antibodies, the humanized variants of SEM120-IIIC1, BLC3 and B2-7 were diluted in 100 μL of 2% M-PBST and incubated in the antigen-coated wells for 1 hr at room temperature. The wells were then washed three times with PBST. Bound scFv-Fc were detected with a peroxidase-labeled goat anti-mouse antibody recognizing the murine part of the Fc fragment (A0168, Sigma-Aldrich). In case of A1HC38, the antigen ELISA was performed with scFv phage particles. The humanized variants of A1HC38 were diluted in 100 μL of 2% M-PBST and incubated in the antigen-coated wells. After 1 hr incubation at room temperature, the wells were washed three times with PBST. Bound scFv phage particles were detected with mouse anti-pIII antibody (PSKAN3, MoBiTec) and a peroxidase-labeled goat anti-mouse antibody (A0168, Sigma-Aldrich). Afterwards, antigen binding was visualized in a detection reaction with TMB (3,3’,5,5’-tetramethylbenzidine) as the substrate. The staining reaction was stopped by adding 100 μL of 1 N sulfuric acid. Absorbance at 450 nm (reference wavelength: 620 nm) was measured in a SUNRISE^™^ microtiter plate reader (Tecan, Männedorf, Switzerland).

### Affinity measurement

The affinity of the germline-humanized antibodies were measured by surface plasmon resonance (SPR) with a Biacore X instrument (GE-Healthcare, Chalfont St Giles, UK). Each antibody (1000 resonance units [RU]) was immobilized on a CM5 chip via amine coupling, according to the manufacturer’s instruction. A flow rate of 30 μL/min was adjusted and six dilutions of BoNT (0.16 μM to 5 nM in HBS-EP buffer) were tested. After each measurement, the chip was regenerated for 30 s with glycine-HCl (pH 1.5) at a flow rate of 10 μL/min. The affinity of the antibodies was calculated with Biaevaluation software [51] and checked in internal consistency tests [52].

### *In vivo* neutralization studies in mouse flaccid paralysis assay

Protection capacities of selected IgGs was assessed in mouse flaccid paralysis assay as previously described [38] and were tested individually and in combination against BoNT/A1 (hu8SEM120-IIIC1 and hu8A1HC38) or BoNT/B2 (hu8BLC3 and hu8B2-7) serotypes of toxin. Purified BoNT/A1 heamagglutinin-free holotoxin (Metabiologics Inc, Madison, Wi. USA) was purchased with specific activity of 2.3 x 108 LD_50_/mg, and 0.4 LD_50_ (1.74 pg) was used per dose. Botulinum B2 (BoNT/B2) complex toxin was prepared by Institut Pasteur (Paris, France) with provided activity of 6.8 x 104 LD_50_/mg (total protein) and 0.2 LD_50_ was used per dose. In both cases, the sub-lethal dose of toxin was selected from the previous studies [53] as the highest dose of toxin inducing local flaccid paralysis within 48 hrs after subcutaneous injection into left inguinocrural region of mice. This selected dose (in range 0.2–0.4 LD_50_) will not cause death or systemic symptoms of botulism. Selected antibodies were studied for protection properties at four or five concentrations ranging from 100 μg to 0.1 μg per dose. For the combination protocol two antibodies against the heavy and light chains of BoNT/A (hu8SEM120-IIIC1 and hu8A1HC38) or BoNT/B (hu8BLC3 and hu8B2-7) were used at a range of concentrations between 50 μg and 0.1 μg per dose of each IgG, and were pre-mixed with the same dose of toxin. All dilutions were prepared in gelatin (0.2% w/v) phosphate (50 mM di-sodium hydrogen orthophosphate) buffer, (GPBS, pH 6.5), and toxin:antibody mixtures were left for 30 min at room temperature before injecting 0.1 mL subcutaneously into female MF1 strain of mice, weighing between 16–20 g (n = 4 per dose). All injections were performed within 30 min and all the mice were injected in the left inguinocrural region. Scores were recorded at 48 hr post injection with intensity of paralysis ranging from 0, (no sign of paralysis), to scores between 1 and 4, defined by an increasing extent of local flaccid paralysis.

Positive control group of mice were injected with BoNT/A1 or BoNT/B2 toxin alone, and negative control group of mice were injected with the maximum concentration of each antibody (50 μg) used in the assay, in the absence of toxin. Assay was considered valid if positive control group of mice showed visible local paralysis effect induced by selected dose of toxin (with a mean score close to 3) and mice receiving highest dose of IgG preparations, in the absence of toxin, did not show any visible paralytic effect.

### *In vivo* protection (lethal assays)

The neutralizing activity of the antibodies was evaluated in the *in vivo* mouse lethality test as previously described [42]. Neutralization tests were performed using crude acid-precipitated toxins from strain Hall (type A1) and strain BL6 (type B2) [23,54]. Toxin preparations were diluted in 50 mM phosphate buffer pH 6.3 containing 0.2% gelatin (PB-G) and calibrated to approximately 10x mouse 50% lethal doses (10 MLD_50_) per mL. Ten-fold and then two-fold serial dilutions of antibodies in PB-G were incubated with 10 MLD_50_ of BoNT type A1 or B2 preparations for 30 min at room temperature. Afterwards, 0.5 mL of the mixtures (5 MLD_50_) were injected by intraperitoneal route into Swiss male mice weighing 20–22 g. Groups of at least 4 mice were used for each dilution. Mice were observed at regular intervals and any symptoms recorded every day for 4 days.

## Supporting Information

S1 Fig Titration ELISA of the humanized variants of A1HC38 against recombinant heavy chain of BoNT/A1. Binding of the germline-humanized anti-BoNT/A1 antibodies (hu1-hu8A1HC38) as scFv phage (2.5x10^5^ up to 5x10^8^ cfu) was tested on 100 ng recombinant BoNT/A1 heavy chain.
(TIFF)

S2 Fig Titration ELISA of the humanized variants of BLC3 against recombinant light chain of BoNT/B2. Binding of the germline-humanized anti-BoNT/B2 antibodies (hu1-hu8BLC3) as scFc-Fc was tested on 100 ng recombinant BoNT/B2 light chain.
(TIFF)

S3 Fig Titration ELISA of the humanized variants of B2-7 against recombinant heavy chain of BoNT/B2. Binding of the germline-humanized anti-BoNT/B2 antibodies (hu1-hu8B2-7) as scFc-Fc was tested on 100 ng recombinant BoNT/B2 heavy chain.
(TIFF)
