# Supplementary figures and images for: Development of Germline-Humanized Antibodies Neutralizing Botulinum Neurotoxin A and B
Source: PLoS One. 2016 Aug 25;11(8):e0161446. doi: 10.1371/journal.pone.0161446 (PMC4999263; doi:10.1371/journal.pone.0161446)

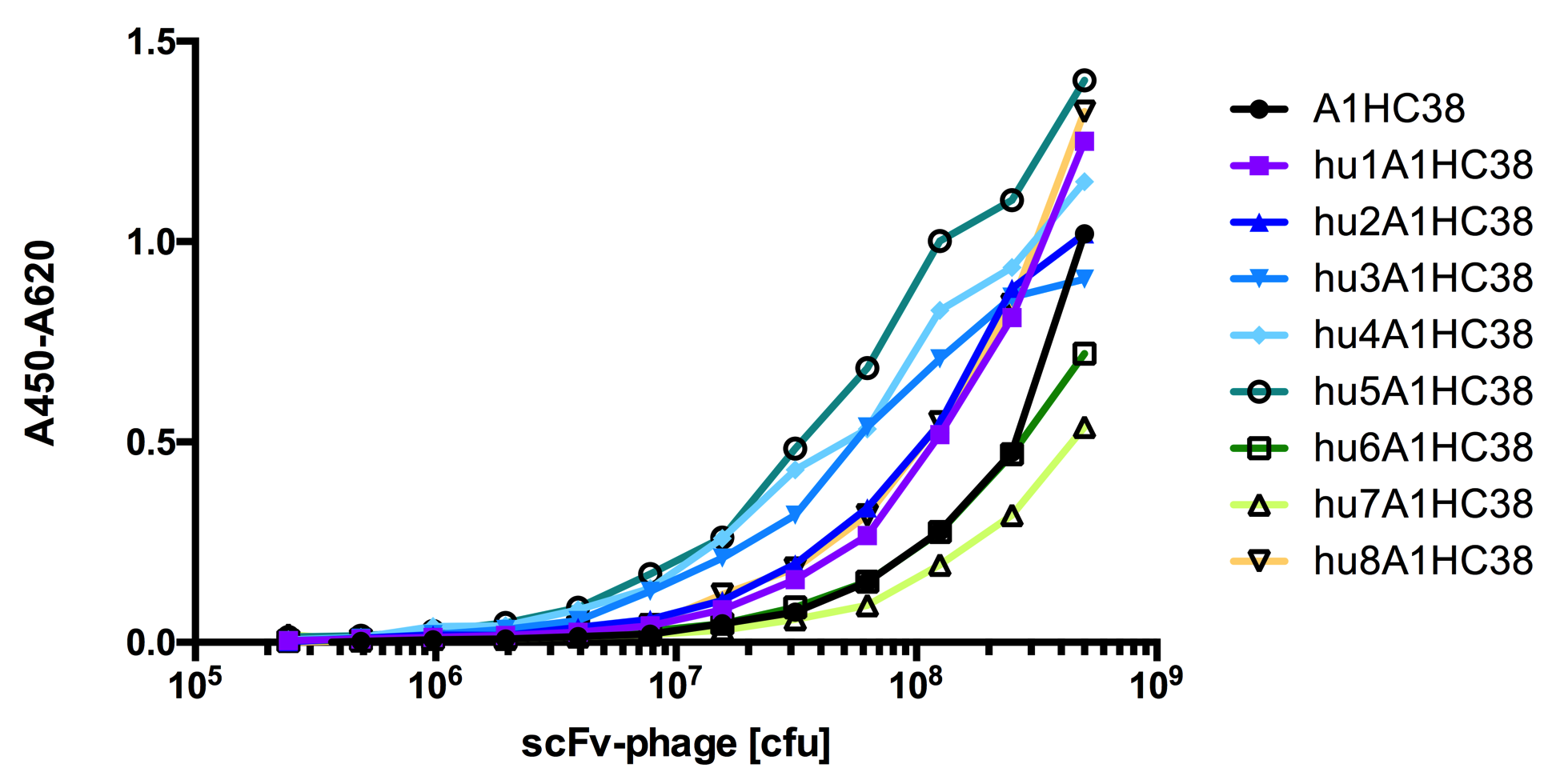

Supplement: S1 Fig — Binding of the germline-humanized anti-BoNT/A1 antibodies (hu1-hu8A1HC38) as scFv phage (2.5x10^5^ up to 5x10^8^ cfu) was tested on 100 ng recombinant BoNT/A1 heavy chain. (TIFF) [file pone.0161446.s001.tiff]

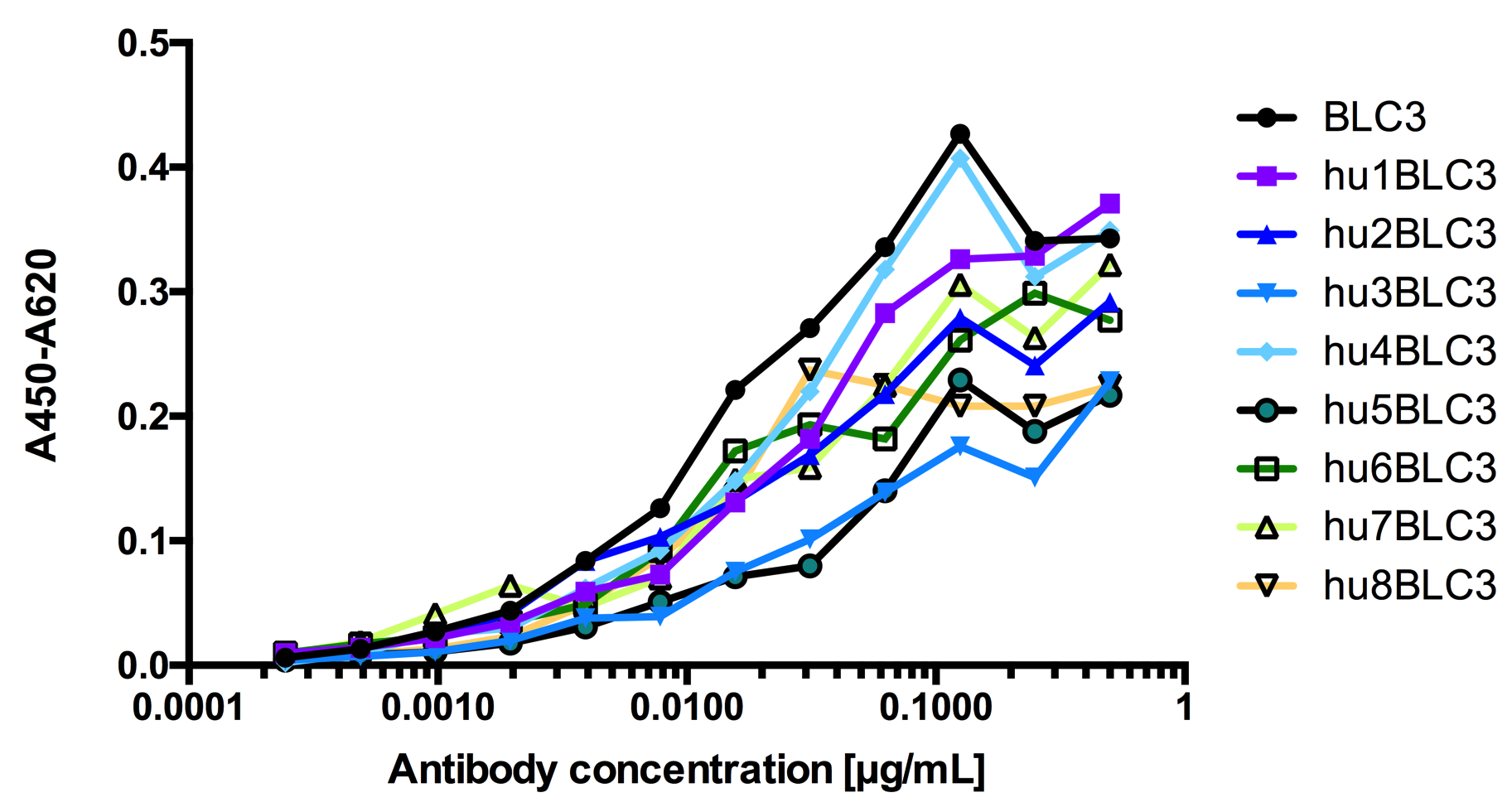

Supplement: S2 Fig — Binding of the germline-humanized anti-BoNT/B2 antibodies (hu1-hu8BLC3) as scFc-Fc was tested on 100 ng recombinant BoNT/B2 light chain. (TIFF) [file pone.0161446.s002.tiff]

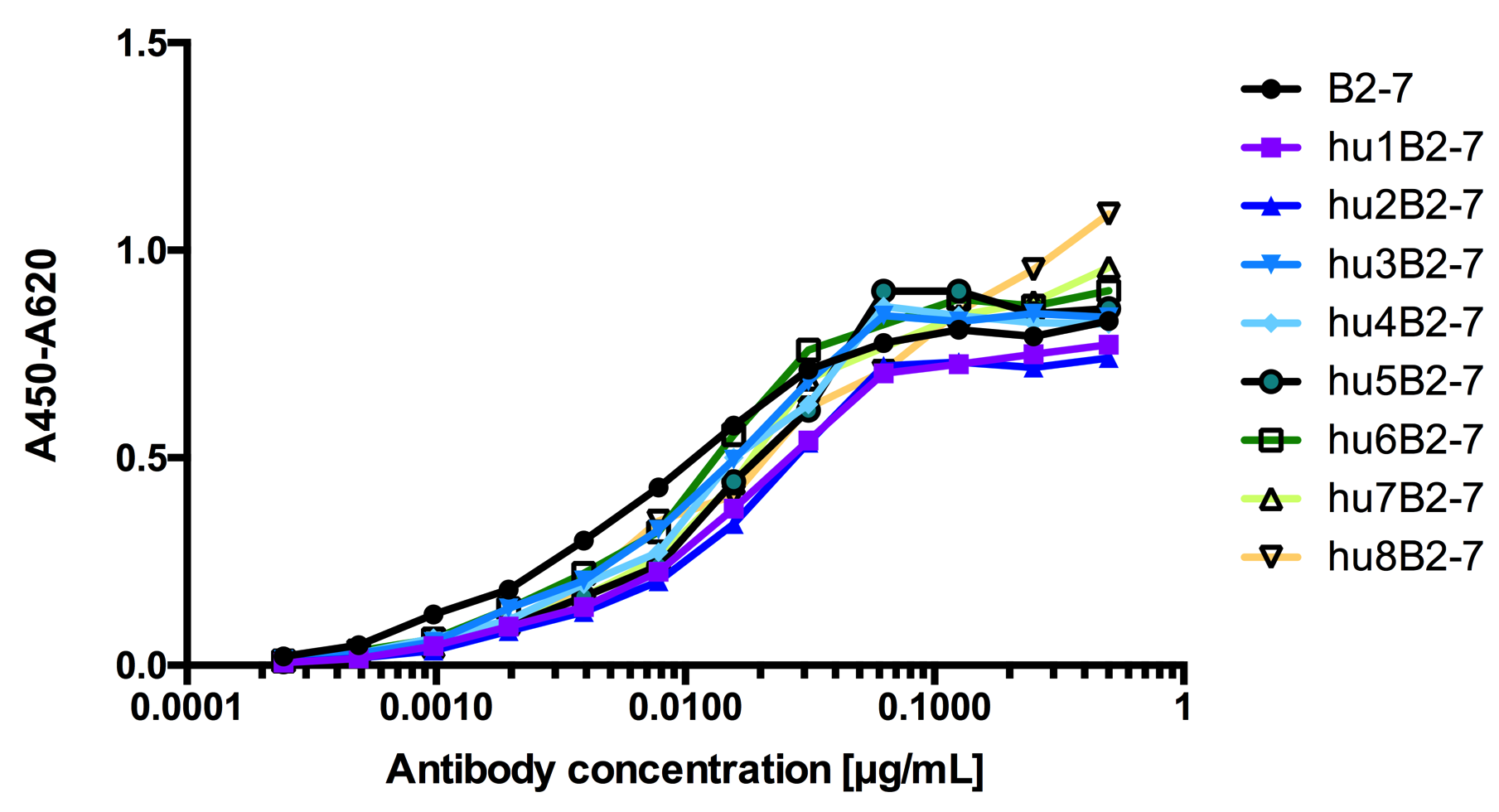

Supplement: S3 Fig — Binding of the germline-humanized anti-BoNT/B2 antibodies (hu1-hu8B2-7) as scFc-Fc was tested on 100 ng recombinant BoNT/B2 heavy chain. (TIFF) [file pone.0161446.s003.tiff]
